# Chlorpheniramine Maleate Throat Spray for the Treatment of COVID-19-Induced Acute Viral Pharyngitis: Case Series

**DOI:** 10.7759/cureus.34310

**Published:** 2023-01-28

**Authors:** Mari L Tesch, Rahul Dasgupta, Uzzam Ahmed Khawaja, Marcos Sanchez-Gonzalez, Rahaghi Franck

**Affiliations:** 1 Research and Development, Dr. Ferrer Biopharma, Hallandale Beach, USA; 2 Pulmonary and Critical Care Medicine, Aventura Hospital and Medical Center, Aventura, USA; 3 Internal Medicine, Jinnah Medical and Dental College, Karachi, PAK; 4 Clinical and Translational Research, Larkin Community Hospital, South Miami, USA; 5 Health Services Administration, Lake Erie College of Osteopathic Medicine, Bradenton, USA; 6 Medicine and Surgery, Cleveland Clinic Florida, Weston, USA

**Keywords:** infectious disease, visual analog scale, chlorpheniramine maleate, acute viral pharyngitis, sars-cov-2

## Abstract

Acute viral pharyngitis (AVP) is a common respiratory illness affecting many individuals. Despite symptomatic treatment management of AVP, therapies are lacking to target a broad spectrum of viruses and the inflammatory nature of the disease. Available for many years, Chlorpheniramine Maleate (CPM), is considered a low-cost and safe first-generation antihistamine displaying antiallergic, anti-inflammatory, and as of recently, identified as a broad-spectrum antiviral agent with activity against influenzas A/B viruses and SARS-CoV-2. Efforts have been made to identify repurposed drugs with favorable safety profiles that could significantly benefit the treatment of COVID-19-induced symptoms. The present case series highlights three patients in which a CPM-based throat spray was used to alleviate the symptoms of COVID-19-induced AVP. The CPM throat spray was associated with significant improvements in patient symptoms after approximately three days of use as opposed to the typical five to seven days reported elsewhere. While AVP is a self-limited syndrome and usually improves without pharmaceutical therapy, CPM throat spray may significantly reduce the overall time that the patient has symptoms. Additional clinical studies to evaluate the efficacy of CPM for the treatment of COVID-19-induced AVP are warranted.

## Introduction

Acute pharyngitis (AP) is a highly prevalent community-acquired infection characterized by a sore throat and inflammation. AP is mainly caused in many cases (30%-60% of the time) by viruses. Moreover, self-limited acute viral pharyngitis (AVP), is generally treated symptomatically, and since it is not a bacterial etiology it does not require antibiotics [1]. The only type of AP that calls for antibiotics involves group A Streptococcus, representing about 10% of all adult cases [2]. Despite decades of efforts to reduce the antibiotic prescription rate for AP, it is currently about 60%, far exceeding what is clinically justified [2]. It is important to note that the overuse of antibiotics for the treatment of AVP could lead to unwanted complications and antibiotic resistance of pathogenic bacterial species representing a public health concern [3]. Hence, effective therapies to target the causative infectious agent, which in this case are viruses, are warranted.

As a viral agent, SARS-CoV-2 may also induce laryngitis and pharyngitis. The Omicron variant seems to be characterized by upper respiratory tract symptoms, including nasal discharge and sore throat. A recent case report highlights a patient complaining of a sore throat and difficulty swallowing saliva that persisted for a day. Evaluation via laryngoscopy revealed severe swelling of the trans glottic region and exudates on the larynx [4]. Therapeutic goals for treating AVP include amelioration of symptoms, decrease in contagion and transmission, and prevention of complications (pneumonia, respiratory failure, and death). Over-the-counter lozenges, sore throat drops, and throat sprays are also available to keep the affected area moisturized or anesthetized [5]. Despite a symptomatic treatment and management of both non-COVID and COVID-19-induced AVP, specific treatments targeting viral and inflammatory causes are lacking.

Available for many years, oral chlorpheniramine maleate (CPM) is considered a low-cost and safe first-generation antihistamine displaying antiallergic, anti-inflammatory, and bronchodilator properties [6-8]. Moreover, CPM has been identified as a broad-spectrum antiviral agent with activity against influenzas A/B viruses and even SARS-CoV-2 [9-11]. Accordingly, the present case series highlights the efficacy of a throat spray formulation based on the antiallergic drug CPM for managing symptoms associated with COVID-19-induced AVP.

## Case presentation

**Figure 1:**
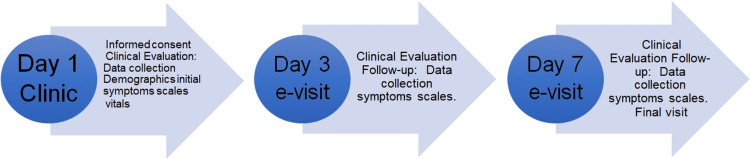
Workflow summary of the procedures Three primary variables: pain on swallowing=POS, urge to cough=UTC, and hoarseness=HO. The five secondary variables: dry mouth and throat=DMT, reddening of the oropharynx=RO, reddening of the larynx=RL, burning sensation in the throat=BST, and patient’s general health condition=GHC.

The present case series comprise three patients who signed informed consent and were screened in a primary care clinic over a period of two weeks. Patients with moderate to severe uncomplicated symptoms of sore throat and pain that worsen with swallowing, urge to cough, and hoarseness was included. The patients were identified by their clinical presentation and diagnosed with AVP by a throat examination conducted by the physician and a PCR or antigen-positive test for SARS-CoV-2. Patients were asked to use CPM 0.5% (ClorRelief^TM^; Dr. Ferrer Biopharma, Hallandale Beach, FL) oral spray administered two sprays (0.2mL each) three to four times a day for a total of 6-8 mg per day for seven days. Figure 1 outlines the workflow summary of the case series. Patients' COVID-induced AP symptom resolution in response to CPM throat spray was accessed using a visual analog scale of 0 (no symptoms) to 10 (severe symptoms).

Case #1

**Figure 2:**
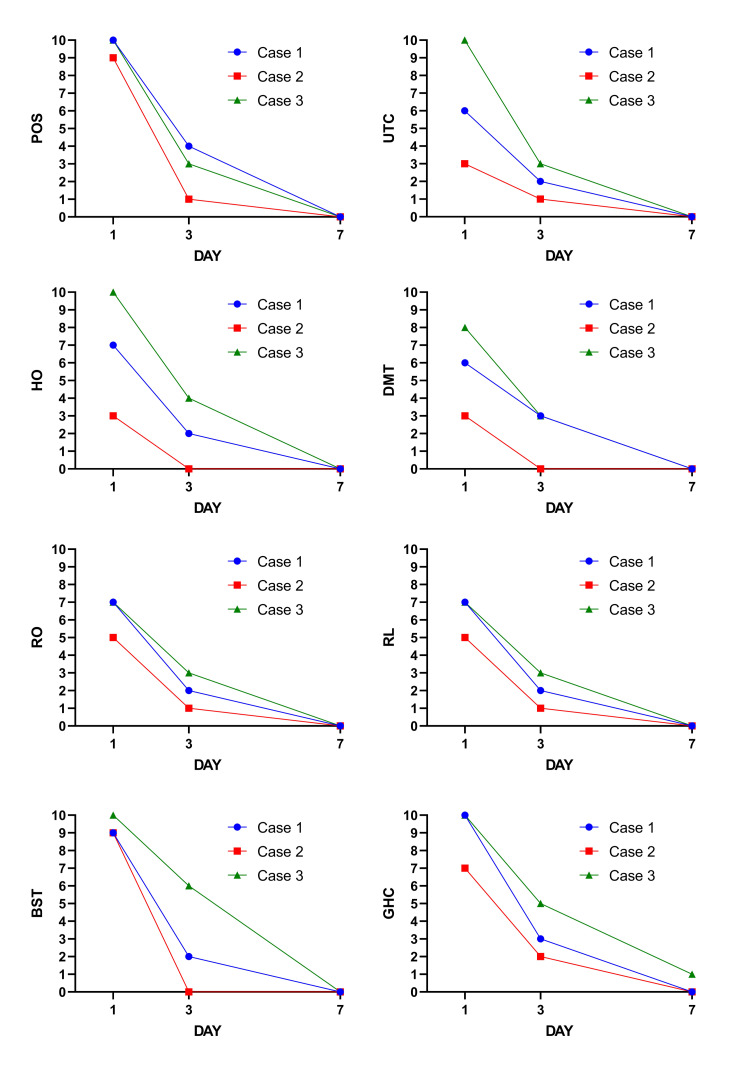
COVID-19-induced acute pharyngitis symptom resolution in response to chlorpheniramine maleate throat spray Notes: Visual analog scale 0 (no symptoms) to 10 (severe symptoms) Three primary variables: pain on swallowing=POS, urge to cough=UTC, hoarseness=HO. Five secondary variables: dry mouth and throat=DMT, reddening of the oropharynx=RO, reddening of the larynx=RL, burning sensation in the throat=BST, and patient’s general health condition=GHC.

A 58-year-old Hispanic woman with a history of chronic rhinitis, diabetes on insulin treatment, squamous cell carcinoma (remission), and sleep apnea using a continuous positive airway pressure (CPAP) device. The patient reported having a high fever, severe muscle and headache pain, sore throat, dry throat with a burning sensation, nasal congestion with postnatal drip, and cough for the last two days. The antigen on the day of the visit was positive for SARS-CoV-2. The patient was administered two doses of the COVID-19 vaccine two months prior. The patient also reported malaise, fever, and fatigue. In addition to CPM oral spray treatment was started with Vitamin C 1000 mg twice a day, zinc 100 mg once a day, Vitamin D3 5000IU once a day, and ibuprofen or acetaminophen as needed, plus daily phone call follow-ups. The patient reported significant improvement in the burning sensation immediately after the spray was applied. On the second day, she felt better as the hoarseness decreased; by day 3 of treatment, the sore throat, hoarseness, and burning had improved by more than 50% (Figure 2). The fever lasted four days, with muscle pain and tiredness persisting slightly until day 5.

Case #2

A 37-year-old Caucasian overweight (BMI of 27) female patient with a history of intermittent allergic rhinit to the outpatient clinic. During the consultation, the patient complained of fever, headache, malaise, sore throat, dryness with a sandpaper-like sensation in the throat, and the feeling of a closed throat. The patient reported difficulty swallowing, no nasal symptoms, and having some coughing but no shortness of breath. The patient reported having had three doses of the COVID-19 vaccine. Treatment was started with CPM oral spray, intranasal saline, Vitamin C 1000 mg twice a day, zinc 100 mg once a day, Vitamin D3 5000IU once a day, and ibuprofen or acetaminophen as needed, and a daily follow-up. The patient reported a drastic improvement in throat symptoms (Figure 2). The fever lasted one day, and the pain and throat symptoms were better by day 3.

Case #3

An 83-year-old male Hispanic patient with a history of controlled diabetes mellitus, hypertension, sleep apnea, and a history of smoking called the outpatient clinic stating that he had a severe headache, sore, dry throat with a burning sensation, a slight cough, and congestion for the last 24 hours. He was on lisinopril for hypertension, using a continuous positive airway pressure (CPAP) device for sleep apnea, and received three doses of the COVID-19 vaccine 40 days prior to study. On presentation to the clinic, a COVID-19 antigen test was performed, which tested positive. The patient-reported experiencing severe headache, sore throat, dry throat with a burning sensation, coughing, and nasal congestion for the last 24 hours. Additionally, the patient reported malaise, fever, and fatigue. Treatment was started with Vitamin C 1000 mg twice a day, zinc 100 mg once a day, Vitamin D3 5000IU once a day, and ibuprofen or acetaminophen as needed, plus daily follow-up. Treatment with oral CPM spray was started. The patient reported significant improvement in the burning sensation immediately after the spray was applied, and by the end of the first day, he felt somewhat better from hoarseness. On day 2, the doctor added fluticasone nasal spray for treatment, but sore throat, hoarseness, and burning improved by more than 50% as reported by the patient. The fever lasted three days, and the muscle pain for about six days. By day 7, the patient recovered (Figure 2).

## Discussion

Through these case series, we intended to examine the potential use of a CPM-based throat spray formulation, a drug with antiallergic, anti-inflammatory, and broad-spectrum antiviral effects, to alleviate the symptoms associated with COVID-19-induced AVP. The present case series included three patients presenting with co-morbidities, including diabetes mellitus, hypertension, allergic rhinitis, and sleep apnea. They were experiencing constitutional COVID-19 symptoms like sore throat, cough, dyspnea, nose plugging with postnasal drip, and a dry throat with a burning sensation. All the patients reported significant improvement in symptoms of COVID-19-induced pharyngitis within the first day of administration and near complete resolution by the third day. These cases and the improvement experienced by the patient provide early evidence to propose the use of the broad-spectrum antiviral CPM as a supported treatment for AVP. Although limited conclusions can be drawn from these case series, the early necessary steps to propose a larger randomized controlled trial to use CPM for treating AVP have been laid out in this work.

In general, it has been documented that the symptoms associated with AVP lasted for five to seven days [12,13]. Recently, a randomized controlled trial conducted by Dao et al. showed that AVP symptoms including a burning sensation in the throat, an urge to cough, dry mouth and throat, and pain in swallowing had a poor improvement over the course of seven days (~30%) in those treated with placebo [14]. In the present case series, patients displayed a substantial improvement with just three days of treatment reporting lower symptoms of AVP corresponding to an improvement of over 60% and complete resolution by day seven. The authors acknowledge that there are certain limitations associated with the nature of the case series such as control which made it difficult to discern between the effects attributable to CPM versus other supporting medications. However, considering that the patients reported a faster relief of symptoms in just three days and no symptoms on day 7 of use cannot be overlooked.

A possible underlying mechanism to explain the improvement in symptoms experienced by the patients may be associated with the ability of CPM to block the primary mediators linked to the inflammatory reactions [6,7] that may contribute to the manifestations seen in allergic rhinitis, which may also contribute to pharyngitis associated with a viral illness [5,15]. In addition, CPM carries broad-spectrum antiviral properties that may contribute by minimizing and neutralizing viral particles [9,11,16]. In an animal study, CPM demonstrated significant inhibitory potential against divergent influenza A strains and one influenza B strain suggesting a broad antiviral spectrum of action [10]. Interestingly other antihistamines may carry the capability for clinical use in managing and preventing infection by the influenza virus and SARS-CoV-2 [16,17].

## Conclusions

In conclusion, the current case series serves as an additional concerted effort to identify repurposed drugs with favorable safety profiles that could significantly benefit the treatment of COVID-19-induced symptoms. Patients using the CPM throat spray reported favorable symptoms resolution to COVID-19-induced pharyngitis. Further double-blind, randomized controlled trials can be carried out to support the current findings and potential implications for the treatment of AVP.
